# Leishmaniasis and tumor necrosis factor alpha antagonists in the Mediterranean basin. A switch in clinical expression

**DOI:** 10.1371/journal.pntd.0007708

**Published:** 2019-08-30

**Authors:** Pau Bosch-Nicolau, Maria Ubals, Fernando Salvador, Adrián Sánchez-Montalvá, Gloria Aparicio, Alba Erra, Pablo Martinez de Salazar, Elena Sulleiro, Israel Molina

**Affiliations:** 1 Department of Infectious Diseases, Hospital Universitari Vall d’Hebron, PROSICS Barcelona, Universitat Autònoma de Barcelona, Spain; 2 Department of Dermatology, Hospital Universitari Vall d’Hebron, Universitat Autònoma de Barcelona, Spain; 3 Department of Rheumatology, Hospital Universitari Vall d’Hebron, Universitat Autònoma de Barcelona, Spain; 4 Department of Clinical Microbiology, Hospital Universitari Vall d’Hebron, PROSICS Barcelona, Universitat Autònoma de Barcelona, Spain; Ohio State University, UNITED STATES

## Abstract

**Background:**

Tumor necrosis factor alpha (TNF-α) blockers are recognized as a risk factor for reactivation of granulomatous infections. Leishmaniasis has been associated with the use of these drugs, although few cases have been reported.

**Methodology:**

We performed a retrospective observational study including patients with confirmed leishmaniasis acquired in the Mediterranean basin that were under TNF-α blockers therapy at the moment of the diagnosis. Patients diagnosed in our hospital from 2008 to 2018 were included. Moreover, a systematic review of the literature was performed and cases fulfilling the inclusion criteria were also included.

**Principal findings:**

Forty-nine patients were analyzed including nine cases from our series. Twenty-seven (55.1%) cases were male and median age was 55 years. Twenty-five (51%) patients were under infliximab treatment, 20 (40.8%) were receiving adalimumab, 2 (4.1%) etanercept, one (2%) golimumab and one (2%) a non-specified TNF-α blocker. Regarding clinical presentation, 28 (57.1%) presented as cutaneous leishmaniasis (CL), 16 (32.6%) as visceral leishmaniasis (VL) and 5 (10.2%) as mucocutaneous leishmaniasis (MCL). All VL and MCL patients were treated with systemic therapies. Among CL patients, 13 (46.4%) were treated with a systemic drug (11 received L-AmB, one intramuscular antimonials and one miltefosine) while 14 (50%) patients were given local treatment (13 received intralesional pentavalent antimonials, and one excisional surgery). TNF-α blockers were interrupted in 32 patients (65.3%). After treatment 5 patients (10.2%) relapsed. Four patients with a CL (3 initially treated with local therapy maintaining TNF-α blockers and one treated with miltefosine) and one patient with VL treated with L-AmB maintaining TNF-α blockers.

**Conclusions:**

This data supports the assumption that the blockage of TNF-α modifies clinical expression of leishmaniasis in endemic population modulating the expression of the disease leading to atypical presentations. According to the cases reported, the best treatment strategy would be a systemic drug and the discontinuation of the TNF-α blockers therapy until clinical resolution.

## Author summary

Tumor necrosis factor alpha (TNF-α) blockers are widely used in numerous inflammatory diseases such rheumatoid arthritis, psoriasis or inflammatory bowel diseases. They have been recognized as a risk factor for reactivation of granulomatous infections. Although few cases have been reported, Leishmaniasis has been associated with the use of these drugs. *Leishmania infantum* is the main causative agent of leishmaniasis in Southern Europe and is prone to produce the visceral form. However, TNF-α has been implicated in the initial events of the infection mediating the disease expression. In our series, we have observed a surprisingly high proportion of cutaneous form (32.6%) and muco-cutaneous form (10.2%). Clinical outcome observed in this series is also unusual. Four cases (14.3%) with cutaneous leishmaniasis who received local therapy relapsed. Among patients with visceral leishmaniasis, one patient who maintained TNF-α blockers therapy relapsed despite etiological treatment. This data supports the assumption that the blockage of TNF-α modifies clinical expression of leishmaniasis leading to atypical presentations. According to the cases reported we proposed as best treatment strategy a systemic drug and the discontinuation of the TNF-α blockers therapy until clinical resolution.

## Introduction

Tumor Necrosis Factor-α (TNF-α) is a crucial cytokine in the inflammatory cascade by activating the type 1 T helper (Th1) immune response, enhancing the activity of the macrophages and essential for the formation and maintenance of granulomas [1].

Since TNF-α has been implicated in numerous immune-mediated disorders, the blockage of this cytokine has been studied as a therapeutic strategy against such diseases. Nowadays, the anti-TNF based therapy is widely used and approved for the treatment of chronic inflammatory conditions as rheumatoid arthritis, polyarticular juvenile idiopathic arthritis, plaque psoriasis and psoriatic arthritis, ankylosing spondylitis and inflammatory bowel diseases [2]. The first approved TNF-α blocker was etanercept (Enbrel) in May 1998 followed by infliximab (Remicade) in November 1999, adalimumab (Humira) in December 2002, certolizumab (Cinzia) in April 2008 and golimumab (Simponi) in April 2009.

Since their first use, the TNF-α blockers were recognized as a risk factor for reactivation of granulomatous infections such as tuberculosis, intracellular infections such as salmonellosis or listeriosis and other opportunistic fungal or viral infections [3].

Leishmaniasis is a parasitic granulomatous infection and it is endemic to South America, South Asia, Africa and South Europe. The protozoon is an obligate intracellular parasite of mononuclear phagocytic system cells. The clinical spectrum of leishmaniasis comprises subclinical (asymptomatic), localized (cutaneous) and disseminated infection (cutaneous, mucosal and visceral). Its clinical expression is determined on one hand by the species and zimodeme of the parasite and on the other hand by host factors and immune response [4].

Leishmaniasis has been associated with the use of TNF-α blockers, but only few cases have been reported in the literature, mainly in the Mediterranean basin [5,6]. We report nine more cases related to the use of TNF-α blockers and systematically review the published cases acquired in the Mediterranean basin. We also analyze their clinical presentation and discuss the relationship with immunomodulatory therapy. Finally, a therapeutic approach is discussed.

## Methods

We carried out a retrospective observational study including patients with a diagnosis of leishmaniasis in its different forms. All patients were under TNF- α blockers and were diagnosed in our center between 2008 and 2018. We also performed MEDLINE search using the terms *Leishmania*, *leishmaniasis*, *TNF-α inhibitors*, *TNF-α blockers*, *anti-TNF-α*, *adalimumab*, *infliximab*, *etanercept*, *certolizumab and golimumab*. No language or time restrictions were applied. A manual search of the references of the selected manuscript was also performed.

In order to be included in the analysis, cases had to be diagnosed based on direct observation of amastigotes and/or positive *Leishmania* polymerase chain reaction (qPCR) of blood, bone marrow or skin samples.

DNA extraction from blood and bone marrow samples was carried out from 110 μl with silica–membrane technology (NucliSens easyMAG. Biomerieux. France) and eluted in 110 μl according to the manufacturer´s instructions. The extraction protocol from skin biopsy was made in a Magcore Compact (RBC Bioscience. Taiwan) and eluted in 100 μl according to the manufacturer´s instructions. A duplex qPCR targeted to kinetoplast minicircle DNA *of Leishmania sp* and human RNase P gene (Taq Man Human RNase P detection reagent; Applied Biosystems) were performed using the primers and probe described previously [7]. Cycling conditions were a first step of 15 minutes at 95°C followed by 45 cycles at 95°C for 15 seconds and 55°C for 1 minute. Amplifications were carried out in a CFX Real-Time PCR detection system (Bio-Rad, Hercules, CA). *Leishmania infantum* identification was performed by restriction fragment length polymorphism of the internal transcribed spacer regions (ITS-RFLP) in a reference laboratory.

Patients were classified based on the clinical form as follows: VL when compatible signs and symptoms and blood or bone marrow samples had a positive culture or *Leishmania* polymerase chain reaction (qPCR). MCL when compatible signs and symptoms and mucose samples had a positive culture or *Leishmania* qPCR. CL when compatible signs and symptoms and cutaneous samples had a positive culture *Leishmania* qPCR. We assessed the patients’ complete medical records: demographic data, underlying disease, TNF- α blockers therapy, specific treatment and outcomes were recorded. Cure was defined as an absence of clinical signs after a minimum follow-up of 1 year after leishmanicidal treatment discontinuation. Unrelated death was established when a death with no relation to the infection or its treatment occurred during follow-up. Relapses were defined as reappearance of clinical signs after treatment discontinuation during the first year of follow-up unless relapse occurring after this period can be microbiologically demonstrated. Those cases without relevant information or not well identified were excluded from analysis.

### Statistical analysis

Categorical variables are expressed as percentages, and numerical data as the mean±SD for variables with a normal distribution or the median (IQR) for those with a skewed distribution. Categorical variables were compared with the chi-square test or Fisher exact test, and continuous variables with the Student *t* or the Mann-Whitney *U* test, depending on distribution. All statistical tests were 2-tailed, and significance was set at P < .05. Statistical analyses were performed using SPSS version 20.0 (SPSS, Inc., Chicago, IL, USA).

### Ethics statement

Due to its retrospective design, oral consent was obtained by phone contact from the included patients. The study was approved by the Ethics Committee of Vall d’Hebron Research Institute.

## Results

A total of 33 publications were retrieved in our search including forty cases that fulfilled inclusion criteria [8–39]. Thus, a total of 49 cases were analyzed including our 9 cases (Table 1). Twenty-seven (55.1%) cases were male and the median age was 55 (range 7–80) years. Twenty-five (51%) patients were under infliximab treatment at the moment of leishmaniasis diagnosis, twenty (40.8%) were receiving adalimumab, two (4.1%) were receiving etanercept, one (2%) golimumab and another one (2%) received a non-specified TNF-α blocker. From greater to lesser frequency, the underlying disease was psoriatic arthritis in twelve (24.5%) cases, rheumatoid arthritis in twelve (24.5%) cases, ankylosing spondylitis in nine (18.4%) cases, Crohn’s diseases in five (10.2%) cases, plaque psoriasis in five (10.2%) cases and rheumatoid arthritis with psoriasis, ulcerative colitis, juvenile idiopathic arthritis, giant cell arthritis, seronegative arthritis and *folliculitis decalvans* in one case each other. All patients were diagnosed in European hospitals and probable place of infection was Spain in thirty-three (67.3%) cases followed by Greece in five (10.5%) cases, Italy in four (8.2%) cases, France in two (4.1%) cases, Malta in two (4.1%) cases, Algeria in two (4.1%) cases and Turkey in one (2%) case.

**Table 1:** Clinical and microbiological characteristics of patients with leishmaniasis associated to TNF-α antagonist treatment in the Mediterranean Basin.

Patient Number	Author, year and reference	Country (Region)	Anti TNF	Other IS	Sex-Age	Disease	Clinical form	Number of lesions	Specie	Sample	Diagnostic	Treatment	Outcome	Comment
1	Romaní-Costa 2004 (8)	Spain (Catalonia)	Infliximab	-	M-55	PA	VL	-	Leishmania sp.	Bone Marrow	DO+	Parenteral Sb	Cure	
2	Fabre 2005 (9)	France (Languedoc-Rousillon)	Infliximab	Azatioprine Corticosteroids	F-53	RA	VL	-	*L*. *infantum* MON-1	Bone Marrow	DO+ PCR+	L-AmB 15.4mg/Kg Anti-TNF Stopped	Cure	
3	Bagalas 2006 (10)	Greece (Central Macedonia)	Etanercept	Cyclosporin Corticosteroids	F-60	RA	VL	-	Leishmania sp.	Bone Marrow	DO+	L-AmB 50mg/Kg	Dead	Death was due to respiratory superinfection
4	Bassetti 2006 (11)	Italy (Liguria)	Adalimumab	MTX Corticosteroids	F-69	RA	VL	-	Leishmania sp.	Bone Marrow	DO+ PCR+	L-AmB 18mg/Kg Anti-TNF Stopped	Cure	
5	Tektonidou 2008 (12)	Greece (Atica)	Infliximab	MTX 12.5mg/w Pred 7.5g/d	M-45	PA	VL	-	Leishmania sp.	Bone Marrow	PCR+	L.Amb 18mg/Kg Anti-TNF Stopped	Cure	
6	De Leonardis 2008 (13)	Italy (Emilia Romagna)	Infliximab	MTX 7.5mg/w AZA (different times)	M-63	PA	VL	-	Leishmania sp.	Bone Marrow	DO+	L-Amb Anti-TNF Stopped	Cure	
7	Garcia-Vidal 2009 (14)	Spain (Catalonia)	Infliximab	MTX Corticosteroids	M-55	AS	VL	-	*L*. *donovani* complex	Bone Marrow	DO+	Parenteral Sb Anti-TNF Stopped	Cure	
8	Jeziorski 2009 (15)	France (Languedoc-Roussillon)	Infliximab	MTX 10mg/w	F-7	JIA	VL	-	*L*. *infantum*	Bone Marrow	DO+	L-Amb 24mg/Kg	Relapse	Relapsed 26months after L-Amb cessation with a MCL form.
9	Xynos 2009 (16)	Greece (Atica)	Infliximab	MTX 10mg/w Corticosteroids	F-71	GCA	VL	-	Leishmania sp.	Bone Marrow	DO+ PCR+	L-Amb 21mg/Kg	Cure	
10	Moreno 2010 (17)	Spain (Valencian Comunity)	Infliximab	MTX 15mg/w Pred 5–10 mg/d	F-72	RA	VL	-	Leishmania sp.	Bone marrow Duodenal biopsy	DO+	L-AmB 21mg/Kg Anti-TNF Stopped	Cure	
11	Moltó 2010 (18)	Spain (Catalonia)	Adalimumab	MTX 20 mg/w Pred 5–10 mg/d	M-60	RA	VL	-	Leishmania sp.	Bone Marrow	DO+	L-AmB 30mg/Kg Anti-TNF Stopped	Cure	
12	Kritikos 2010 (19)	Greece (Atica)	Infliximab	-	F-77	RA	VL	-	Leishmania sp.	Bone Marrow	DO+	L-AmB Anti-TNF Stopped	Cure	
13	Erre 2010 (20)	Italy (Sardinia)	Adalimumab	MTX 10mg/w	F-71	RA	VL	-	Leishmania sp.	Bone Marrow	DO+	L-Amb Anti-TNF Stopped	Cure	
14	Khan 2010 (21)	Malta	Adalimumab	MTX	F-74	PA	VL/CL	-	*L*. *infantum*	Bone Marrow Skin biopsy	DO+ PCR+	L-Amb 30mg/Kg Intralesional Sb Anti-TNF Stopped	Cure	Episode considered as visceralization of a CL form
15	Besada 2013 (22)	Spain (Valencian Comunity)	Etanercept	MTX 20mg/w	F-80	PA	VL	-	*L*. *infantum*	Bone Marrow	DO+ PCR +	L-AmB 21mg/Kg Anti-TNF Stopped	Cure	
16	Berry 2013 (23)	Spain (Andalusia)	Adalimumab	MTX 20mg/w	F-69	RA	VL	-	*L*. *infantum*	Bone Marrow Spleen Biopsy	DO+ PCR+	L.AmB 40mg/Kg Anti-TNF Stopped	Cure	
17	García-Castro 2011 (24)	Spain (Andalusia)	Infliximab	MTX 15mg/w Pred 2.5mg/d	F-66	RA	MCL	1	*L*. *Infantum* MON-183	Mucosa biopsy	DO+ Culture +	L-AmB/Sb Anti-TNF Stopped	Cure	
18	García-González 2012 (25)	Italy (Tuscany)	Adalimumab	MTX 10mg/w M-pred 4mg/d	M-49	AS	MCL	1	*L*. *infantum*	Mucosal biopsy	DO+ Culture+	L-AmB 15mg/Kg Anti-TNF Stopped	Cure	
19	Neumayr 2013 (26)	Spain (Balearic Islands)	Adalimumab	-	M-53	PA	MCL	1	*L*. *infantum*	Skin biopsy	PCR+	Miltefosine	Cure	
20	Juzlova 2014 (27)	Turkey/ Croatia	Infliximab	MTX 15mg/w	M-44	CD	MCL	1	*L*. *infantum*	Perianal Biopsy	DO+ PCR+	Intramuscular MgA	Dead	Death was due to fatal arrhythmia as side effect of antimony
21 (Fig 2)	Bosch-Nicolau 2019	Spain (Catalonia)	Infliximab	-	M-53	PA	MCL/VL	1	*L*. *Infantum*	Mucosal biopsy Bone marrow	DO +(biopsy) PCR +	L-AmB 40mg/Kg Anti-TNF Stopped	Cure	Episode considered as a reactivation of a previous CL occurred 4 years before
22	Baltà-Cruz 2009 (28)	Spain (Catalonia)	Infliximab followed Adalimumab	MTX 15mg/w Pred 3.75 mg/d	F-56	RA	CL	1	*L*. *infantum*	Skin Biopsy	DO+ PCR+	Intralesional Sb Surgery	Relapse	MC form treated with L-AmB/ IM Sb. Anti-TNF Stopped Finally cured
23	Mueller 2009 (29)	Spain (Balearic Islands)	Infliximab	-	M-31	AS	CL	1	*L*. *Infantum* MON 1	Skin Biopsy	DO+ PCR+ Culture +	Miltefosine Anti-TNF Stopped	Relapse	C form treated with intralesional Sb. Anti-TNF Stopped Finally cured
24	Schneider 2009 (30)	Algeria	Adalimumab	MTX	F-51	AS	CL	3	*L*. *infantum*	Skin Biopsy	PCR+	L-AmB 21mg/Kg Anti-TNF Stopped	Cure	
25	Xynos 2009 (16)	Greece (Atica)	Infliximab	MTX 10mg/w	M-55	AS	CL	Several	Leishmania sp.	Skin Biopsy	DO+	L-AmB 21mg/Kg Anti-TNF Stopped	Cure	Presented several crusted lesions on face
26	Hakimi 2010 (31)	Algeria / France (Languedoc-Rousillon)	Infliximab	-	M-50	AS	CL	3	*L*. *infantum* MON-1	Skin Biopsy	DO+ PCR+ Culture +	Intralesional MgA Anti-TNF Stopped	Cure	
27	Romero-Maté 2013 (32)	Spain (Madrid)	Adalimumab	-	F-61	RA	CL	6	*L*. *infantum*	Skin biopsy	PCR+	None	-	Lost to follow-up
28	Romero-Maté 2013 (32)	Spain (Madrid)	Adalimumab	MTX 20mg/w	F-59	PA	CL	1	*L*. *infantum*	Skin biopsy	PCR+	Intralesional MgA	Cure	
29	Hernández-Torres 2013 (33)	Spain (Murcia)	Infliximab	-	M-50	P	CL	1	Leishmania sp.	Skin biopsy	DO+ PCR+	L-AmB 40mg/Kg Anti-TNF Stopped	Cure	
30	Català 2014 (34)	Spain (Catalonia)	Infliximab	Leflunomide 20mg/d Pred 5mg/d	M-33	PA	CL	1	Leishmania sp.	Skin biopsy	DO+ Culture +	Intralesional MgA + Cryotherapy Anti-TNF Stopped	Cure	
31	Micallef 2014 (35)	Malta	Adalimumab	MTX	F-45	SNA	CL	1	Leishmania sp.	Skin biopsy	PCR+	Parenteral Sb Anti-TNF Stopped	Cure	
32	Català 2015 (36)	Spain (Catalonia)	Adalimumab	-	F-59	RA	CL	1	Leishmania sp.	Skin biopsy	DO+	L-AmB 40mg/Kg Anti-TNF Stopped	Cure	
33	Marcoval 2017 (37)	Spain (Catalonia)	Adalimumab	-	M-35	CD	CL	1	Leishmania sp.	Skin biopsy	PCR+	Intralesional MgA	Relapse	L-Amb Finally cured
34	Marcoval 2017 (37)	Spain (Catalonia)	Infliximab	-	F-55	CD	CL	1	Leishmania sp.	Skin biopsy	PCR+	Intralesional MgA	Relapse	L-Amb Finally cured
35	Alcover 2018 (38)	Spain (Balearic Islands)	Not specified	-	M-55	RA+P	CL	12	*L*. *infantum*	Skin biopsy	DO+ PCR+ Culture+	L-Amb 40mg/Kg Anti-TNF Stopped	Cure	Presented numerous skin ulcers
36	Martínez-Doménech 2019 (39)	Spain (Valencian Comunity)	Adalimumab	-	F-34	P	CL	1	Leishmania sp.	Skin biopsy	DO+	Intralesional MgA Anti-TNF Stopped	Cure	
37	Martínez-Doménech 2019 (39)	Spain (Valencian Comunity)	Adalimumab	-	M-46	AS	CL	1	Leishmania sp.	Skin biopsy	DO+ PCR+	Intralesional MgA Anti-TNF Stopped	Cure	
38	Martínez-Doménech 2019 (39)	Spain (Valencian Comunity)	Adalimumab	-	F-40	P	CL	1	Leishmania sp.	Skin biopsy	DO+ PCR+	Intralesional MgA	Cure	
39	Martínez-Doménech 2019 (39)	Spain (Valencian Comunity)	Adalimumab	MTX	M-71	PA	CL	2	Leishmania sp.	Skin biopsy	DO+ PCR+	Intralesional MgA Anti-TNF Stopped	Cure	
40	Martínez-Doménech 2019(39)	Spain (Valencian Comunity)	Adalimumab	-	M-53	P	CL	1	Leishmania sp.	Skin biopsy	PCR+	Intralesional MgA Anti-TNF Stopped	Cure	
41	Martínez-Doménech 2019(39)	Spain (Valencian Comunity)	Adalimumab	-	M-22	FD	CL	2	Leishmania sp.	Skin biopsy	DO+ PCR+	Intralesional MgA	Cure	
42 (Fig 1)	Bosch-Nicolau 2019	Spain (Catalonia)	Infliximab	-	M-42	AS	CL	4	*L*. *Infantum*	Skin biopsy	DO+ PCR +	L-AmB 20mg/Kg Anti-TNF Stopped	Cure	Presented numerous skin ulcers
43	Bosch-Nicolau 2019	Spain (Catalonia)	Infliximab	Deflazacort 6mg/d	M-67	AS	CL	1	Leishmania sp.	Skin biopsy	DO+	Surgery	Cure	
44	Bosch-Nicolau 2019	Spain (Catalonia)	Infliximab	Azatioprine	F-54	CD	CL	1	Leishmania sp.	Skin biopsy	DO+ PCR +	Intralesional MgA	Cure	
45	Bosch-Nicolau 2019	Spain (Catalonia)	Golimumab	-	M-71	PA	CL	1	Leishmania sp.	Skin biopsy	DO+	L-AmB 20mg/Kg	Cure	
46	Bosch-Nicolau 2019	Spain (Catalonia)	Infliximab	-	M-67	UC	CL	1	Leishmania sp.	Skin biopsy	DO+	L-AmB 24mg/Kg Anti-TNF Stopped	Cure	
47	Bosch-Nicolau 2019	Spain (Catalonia)	Infliximab	-	M-50	PA	CL	2	Leishmania sp.	Skin biopsy	PCR+	L-AmB 20mg/Kg	Cure	
48	Bosch-Nicolau 2019	Spain (Catalonia)	Infliximab	Azatioprine	M-42	CD	CL	1	Leishmania sp.	Skin biopsy	DO+	L-AmB 16mg/Kg Anti-TNF Stopped	Cure	L-AmB discontinued after 4 doses
49	Bosch-Nicolau 2019	Spain (Catalonia)	Adalimumab		M-74	P	CL	2	Leishmania sp.	Skin biopsy	PCR+	L-AmB 20mg/Kg Anti-TNF Stopped	Cure	

**MTX**: Metrotexate, **Pred**: prednisone, **M-Pred**: Metylprednisolone, **M**: male, **F**: female, **PA**: Psoriatic Arthritis, **RA**: Rheumatoid Arthritis, **AS**: Ankylosing Spondylitis, **JIA**: Juvenile Idiopathic Arthritis, **SNA**: Sero-Negative Arthritis, **GCA**: Giant cell arteritis, **CD**: Crohn’s disease, **UC**: Ulcerative Colitis, **P**: Psoriasis, **FD**: Foliculitis decalvans, **VL**: visceral leishmaniasis, **CL**: cutaneos leishmaniasis, **MCL**: mucucutaneous leishmaniasis, **DO**: Direct Observation, **PCR**: Polymerase Chain Reaction, **Sb**: Sodium tibogluconate, **MgA**: Meglumine antimoniate; **L-AmB**: Liposamal Amphotericine B, **TNF**: Tumor necrosis Factor.

**Fig 1:**
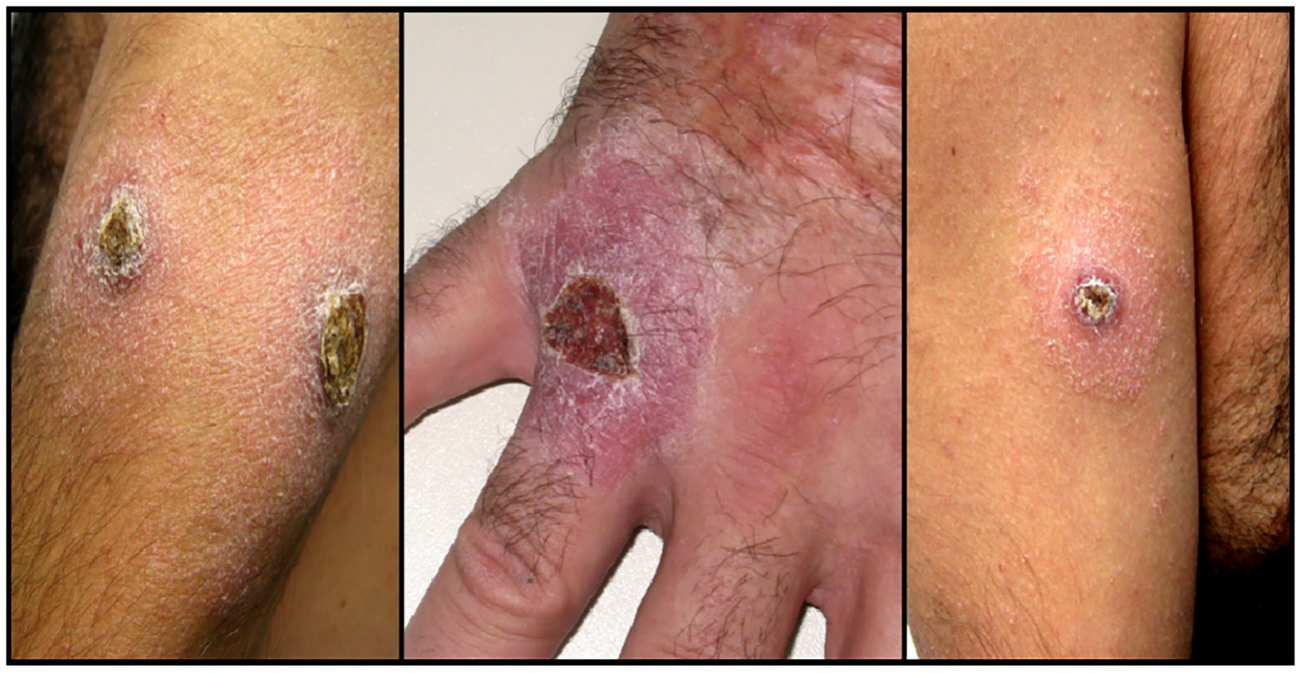
Patient with multiple cutaneous leishmania lesions.

**Fig 2:**
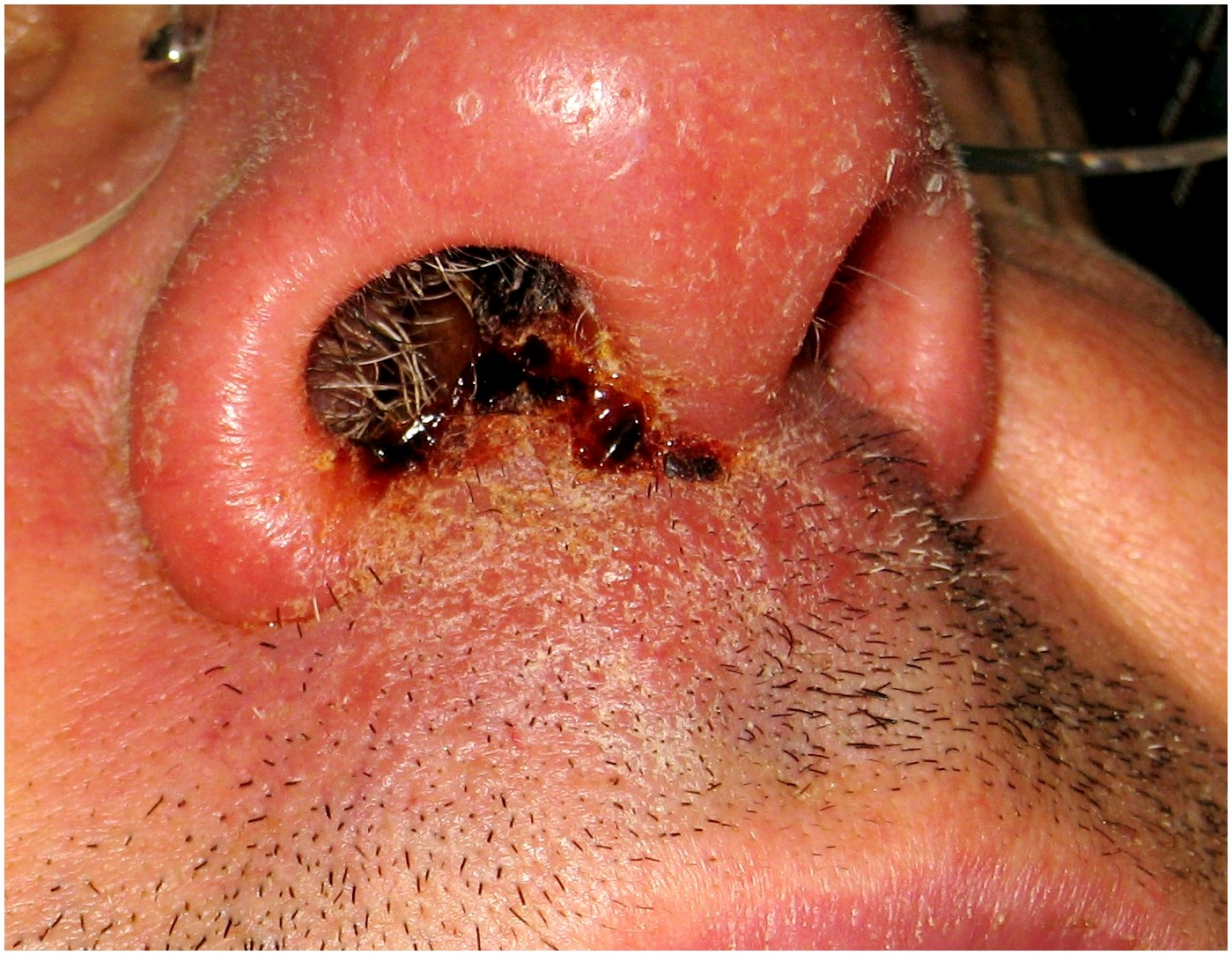
Patient with a mucocutaneous leishmaniasis.

Regarding the clinical presentation, CL was the most frequent form (28 patients, 57.1%), eighteen cases presenting as a solitary ulcerative lesion and ten cases including one attended in our hospital who presented multifocal lesions (Fig 1). Sixteen (32.6%) patients presented VL and five (10.2%) patients had MCL, three in the nasal cavity (Fig 2), one as hyperplasic lesions around perianal mucosa and one as an infiltrative tumor involving upper lip, hard palate and nasal septum. Bone marrow aspirate was performed in one patient with MCL and one patient with CL. Although no amastigotes forms were observed, the patient with MCL had a positive *Leishmania* RT-PCR on the bone marrow sample. Identification to the level of species could be performed in eighteen patients. *L*. *infantum* was identified in seventeen cases and one was reported as *L*. *donovani complex*.

All MCL and VL patients were treated with a systemic therapy. Fifteen (71.4%) of them were treated with liposomal amphotericine B (L-AmB), including one patient treated in combination with intralesional pentavalent antimonials, three (14.3%) patients were treated with parenteral pentavalent antimonials and one (4.8%) patient with miltefosine. Among CL cases, thirteen (46.4%) patients received a systemic treatment; eleven were given L-AmB, one intramuscular antimonials and one miltefosine. Local treatment was given to fourteen (50%) patients, thirteen received intralesional pentavalent antimonials, one combined with surgery and another with cryotherapy and one patient was treated with surgical excision of the lesion. Only one patient defaulted and did not receive any treatment.

**Fig 3:**
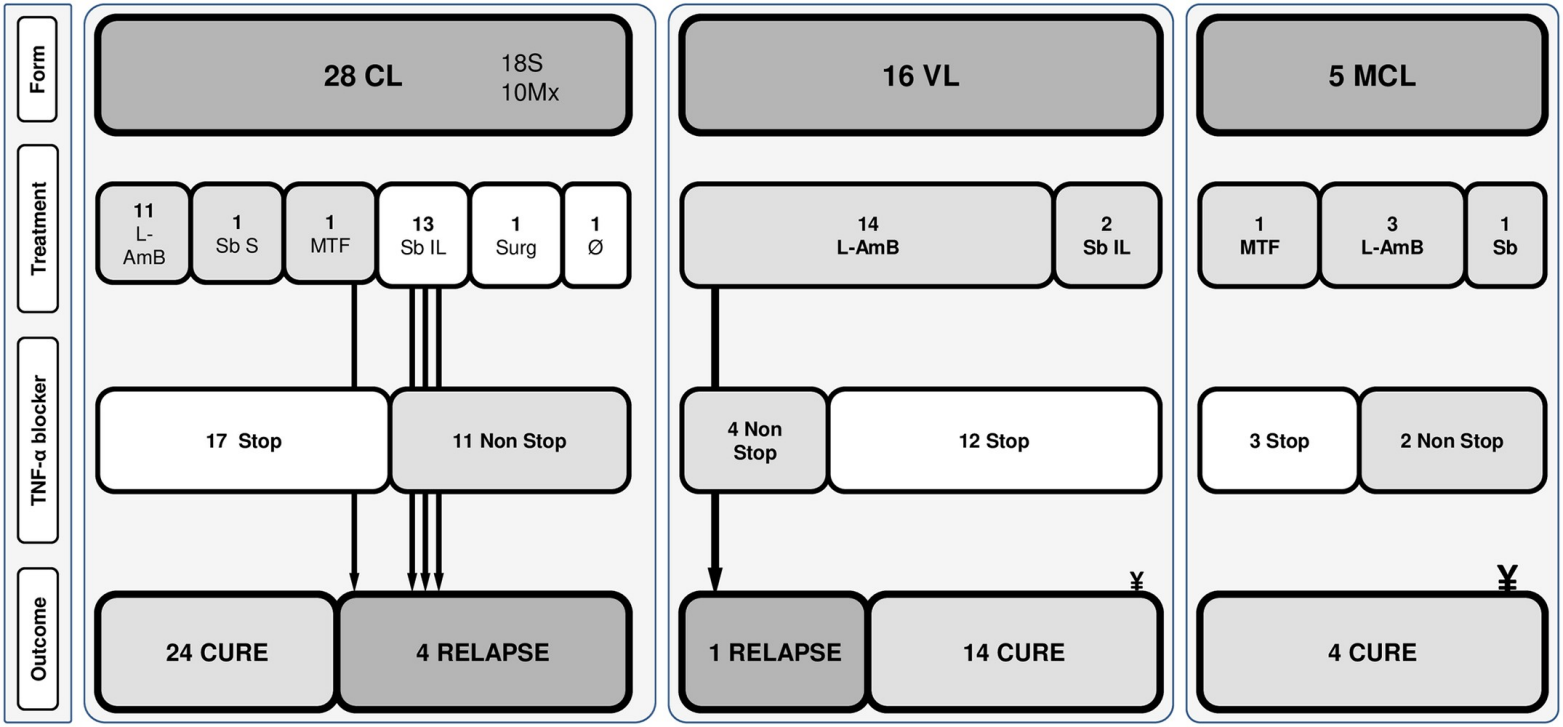
Leishmaniasis form, treatment and outcomes of all included patients. **CL**: cutaneous leishmaniasis, **S**: solitary lesion; **Mx**: multiple lesions; **L-AmB**: liposomal amphotericine-B; **Sb S**: systemic sodium tibogluconate; **MTF**: miltefosine; **Mg IL**: intralesional meglumine antimoniate; **Surg**: surgery; **TNF**: tumor necrosis factor; **Ø** no treatment, **¥** deceased. Arrows represent patients who relapsed and its path shows patient initial treatment and TNF-alpha blocker management.

In thirty-two (65.3%) cases TNF-α blockers therapy was interrupted. After treatment, four (8.2%) patients with CL diagnosis relapsed. Three of these cases were initially treated with local medication and anti-TNF-α was not stopped. After relapse, the three patients received systemic treatment and anti-TNF-α therapy was discontinued in one patient, all achieving clinical cure. Another relapsing patient was initially treated with miltefosine and finally cured with local antimonials therapy. TNF-α blocker therapy was discontinued in both treatment courses. The last relapsing patient was a VL treated with L-AmB and TNF-α blockers were not stopped as her rheumatic disease was active presenting a MCL form 20 months after. When comparing clinical cure of CL patients, although statistical significance could not be reached cure ratios were 92.3% vs. 78.6% (p = 0.6) when patients received systemic treatment or not and 94.1% vs 70% (p = 0.13) when TNF-α blockers therapy could be stopped or not. Finally, the patient who did not receive any treatment was lost to follow up. Two (4.1%) patients died: one patient after a bacterial superinfection in relation to his immunosuppression and the other patient as a result of a fatal arrhythmia during his treatment with systemic antimonials. Case presentation, treatment and outcomes are represented in Fig 3.

## Discussion

According to the published data, TNF-α blockers based therapy seems to be associated with a higher risk of infections, at times with a worse outcome [3]. Nevertheless their efficacy in the management of many chronic inflammatory diseases has reaffirmed its use, representing one of the major breakthroughs in the treatment of these diseases [40].

*L*. *infantum* is the major causative agent of leishmaniasis in Southern Europe and the most frequent zymodeme is MON 1 [41]. Even though it has been suggested that there are dermatotropic and viscerotropic strains, *L*. *infantum* is prone to produce the visceral form of disease and less frequently the cutaneous form. Although different gens have been proposed to contribute to the viscerlalization process, its mechanisms still remains unclear due to the lack of good animal models [42].

It is also well known that in *L*. *infantum* endemic areas there is a frequent natural challenge to *Leishmania* parasites, producing circulating parasitemia in the host during an undefined non-permanent time-span. Some authors have referred to this subclinical form as “asymptomatic carriage”. It means that these patients without symptoms have parasite load only detectable by highly sensitive methods such as RT-PCR [43,44].

In southern Europe the prevalence of asymptomatic carriers is remarkably variable (from 0.5% to 48%) depending on the test used for detection [45]. Those data may suggest that there are natural and frequent challenges between host and parasite in endemic areas, and that an effective immune system is required to control clinical expression.

While it seems that in mammalian hosts *Leishmania* species may be able to infect and develop within non-hematopoietic cells such as fibroblasts it is mostly inside monocyte/macrophage lineage cells where the parasite replicates and develops long-time survival [46]. Since these cells have a central role in both innate response and acquired immune response as parasite antigen presenting, *Leishmania* interferes in an extremely complex manner with the host immunological response [47].

The role of TNF-α in the pathogenesis of the disease has been widely studied in both humans and animal models. As innate TNF-α dependant mechanisms drive cell mediated immunity by activation of CD4+ and CD8+ T cells, TNF-α is known to be fundamental in the initial control of the infection together with other cytokines such as IL-12 and IFN-γ [48]. These cytokines mediate the development of an effective CD4+ Th1 response which is critical to control the infection [49,50]. Furthermore, TNF-α and IFN-γ are responsible for activating leishmanicidal activity of macrophage which is characterized by an increased production of free oxygen radicals and nitric oxide (NO) as well as inducing infected cells apoptosis [51,52].

In CL, a polarized Th1 response with low Th2 cytokines has been related to infection resistance and disease outcome. Besides, Th1 and Th2 pathways may also modify disease expression [53]. However, in patients presenting disseminated diseases such as VL, immune response seems to be a net suppressive-type response. Rather than an inert Th1 type response, a Th2 / Th1 type response associated with the production of down-regulating cytokines such as IL-10 and TGF-β seems to be responsible of the persistent inflammation, the hallmark of clinical leishmaniasis [54,55]. Moreover, regulatory T- and Th17- cells appear to play an important role in susceptibility and disease resistance [56].

Hence, TNF-α has been implicated in the initial events of the infection, in direct leishmanicidal activity and thus controlling the multiplication of the parasite, developing effective acquired immunity for long term control of the disease as well as mediating the disease expression [47].

The classical VL in Mediterranean countries has been found primarily in children and HIV/AIDS infected patients with poor immune status. Nowadays VL cases in adults are also diagnosed among non-VIH immunosuppressed patients [57]. Moreover, CL is less frequent than VL in some Mediterranean countries as Spain although it may be underreported [58]. Besides, MCL is considered exceptional in the Old World [43]. Anyhow, in our series the proportion of CL and MCL it is surprisingly high.

Clinical outcome observed in this series is unusual. The natural course of CL caused by *L*. *infantum* is benign and trends to heal spontaneously. In fact, if lesions are less than 5 cm in diameter and are localized in areas where there is no risk of disfiguring or disabling, only a local wound care is recommended [59]. Four out of twenty-eight (14.3%) cases of CL reported in this series relapsed despite etiological treatment.

Despite of the natural viscerotropism of *L*. *infantum*, the main acquisition of this infection is through the skin. Besides, in endemic areas there is a high exposure to *Leishmania*. Although some studies report up to 48% of prevalence [60], there are only limited cases of CL reported. Consequently, it may be assumed that within immune-competent individuals, the immunity of the host is capable to control the infection, even before that it causes any clinical symptoms.

The reactivation of a latent parasitic form from a granulomatous lesion could be a feasible explanation for those cases. Clinical data from patient number 21 supports this assumption. This patient was diagnosed with a MCL form (Fig 2) 4 years after a CL in his left ankle demonstrated by PCR amplification for *Leishmania* kinetoplastid sequences which healed spontaneously. Because of the latency between the first episode and the second one and the appearance of a distant lesion far from the initial one, the episode was thus considered as a reactivation in the context of the TNF-α blockers therapy.

Previous series including patients with Old and New world leishmania species showed no consensus regarding treatment in patients under TNF-α blockers [26]. In this series only including leishmaniasis acquired at Mediterranean basin countries, besides all patients with VL and MCL were given a systemic therapy, treatments differ considerably. Seventeen (80.9%) patients received L-AmB at different doses ranging from 25.4mg/Kg to 50mg/Kg divided in 5 to 10 doses, three patients received pentavalent antimonials and one miltefosine. L-AmB is usually recommended as first-choice treatment of VL offering similar cure-rates than pentavalent antimonial [61]. Especially, considering that pentavalent antimonials present a worse toxicity profile and major toxicities such cardiac arrhythmias or prolonged QTc interval that can lead to death as in case number 20 of our series [62]. Although miltefosine has successfully been used treating VL, when it comes to *L*. *infantum*, a growing number of treatment failures have been published so further investigation is needed before its systematic recommendation [63]. Concerning CL treatment, approach disparities are even greater. Half patients received systemic treatment with different therapies and dose disparity and the other half received local treatment.

As mentioned before, TNF-α plays a key role in the process of controlling infection that ranges from limiting the replication of the parasite up to eliciting an effective adaptative response. TNF-α blockers therapy could modulate the immunological response to a less effective control of the parasite, hence allowing the infection reactivation or more evident disease expression of newly acquired infections. To this end, patients under treatment with TNF-α blockers have less effective immune mediated mechanisms to control and eradicate a parasite challenge, ending in a higher chance to develop classical and non-classical *L*. *infantum* presenting forms, that otherwise it would have caused a transient parasitemia or an aborted local replication. Likewise, the course and outcome of the disease may be directly affected by the inhibition of TNF- α. Therefore, discontinuation of the anti TNF-α therapy seems to play an important role in the treatment success. Although miltefosine is an excellent option for the New World CL, evidence is scarce for the treatment of *L*. *infantum* infection [64]. Thus, it seems appropriate to limit its use when other treatment options are not available or have failed.

According to the cases reported, in our opinion the best treatment strategy would be a systemic treatment and the discontinuation of the TNF-α blockers therapy. L-AmB has proved to have the best safety profile and compelling evidence of its efficacy in immunosuppressed patients. One of the major concerns is the reintroduction of TNF-α blockers. Taking into account published information, it seems reasonable reintroducing them once clinical cure has been achieved ensuring close clinical follow-up and blood RT-PCR. Although there is scarce information for its recommendation, etanercep or certolizumab have been suggested as a therapeutical option instead of re-introducing other anti-TNF-α monoclonal antibodies due to its possible lower risk of reactivating leishmaniasis [65–66].

This study has the limitations of any retrospective review study. Although is one of the biggest series published on this topic, the scarce number of patients analyzed limits the strength of the recommendations. We agree with existing reports on the fact that despite of not being a common disease in our area, leishmaniasis is likely underreported [58]. Finally, with the results extracted from this study it is not possible to assess the risk of developing clinical leishmaniasis during anti TNF-α therapy. However, there is enough supporting data for the biological plausibility of the influence of the use of such therapy in the appearance of new cases of clinical leishmaniasis with a switch in the disease expression and outcome. The blockage of TNF-α could determine the incapacity to control and eradicate the parasite within the granuloma playing an important role in increasing the risk of progression to clinical disease.

### Conclusions

The increase in the use of TNF-α antagonist has been associated with the emergence of new cases of leishmaniasis. The blockage of TNF-α favors the reactivation of latent leishmaniasis modulating its expression and worsening its clinical outcome.

Once the leishmaniasis is confirmed, systemic drug treatment and the discontinuation of the TNF-α blockers therapy until clinical recovery seems to be the best therapeutic approach when possible.

Otherwise, those patients receiving such therapy and coming from endemic areas require a close monitoring in order to detect early forms and start adequate treatment.

Prospective studies and more participation on declaring existing cases in the adverse events notification system is required in order to assess the risk of leishmaniasis and other opportunistic diseases related to the use of anti TNF-α treatment more accurately.

## Supporting information

S1 Checklist STROBE checklist. (DOCX)
